# How do youth with Delayed Sleep-Wake Phase Disorder experience a chronobiological treatment protocol? An interview study

**DOI:** 10.3389/frsle.2025.1555160

**Published:** 2025-06-13

**Authors:** Ane Wilhelmsen-Langeland, Ingrid Dundas, Ståle Pallesen, Bjørn Bjorvatn, Inger Hilde Nordhus, Ingvild West Saxvig

**Affiliations:** ^1^Bjørgvin District Psychiatric Center, Division of Psychiatry, Haukeland University Hospital, Bergen, Norway; ^2^Department of Health and Caring Sciences, University of Applied Sciences in Western Norway, Bergen, Norway; ^3^Department of Clinical Psychology, University of Bergen, Bergen, Norway; ^4^Department of Psychosocial Science, University of Bergen, Bergen, Norway; ^5^Norwegian Competence Center for Sleep Disorders, Haukeland University Hospital, Bergen, Norway; ^6^Department of Global Public Health and Primary Care, University of Bergen, Bergen, Norway

**Keywords:** circadian rhythm sleep disorders, Delayed Sleep-Wake Phase Disorder, bright light therapy, melatonin, sleep scheduling, interview study

## Abstract

**Objectives:**

Treatment with timed bright light and exogenous melatonin has been shown to effectively advance the sleep-wake rhythm in patients with Delayed Sleep-Wake Phase Disorder (DSWPD). However, the treatment protocol is demanding, which may negatively affect treatment adherence. The objective of the present study was thus to explore how young adults participating in a treatment study for DSWPD experienced the treatment protocol.

**Method:**

Individual semi-structured interviews were conducted with 11 young adults with DSWPD. An introductory request was to rate whether they thought the treatment was worth the effort, with response options ranging from 0 to 100 (totally worth the effort). Interviews were analyzed using thematic analysis.

**Results:**

The mean rating of the treatment was 72.5 (range 60–100), indicating that all participants considered the benefits to outweigh the effort. The thematic analysis resulted in three themes: (1) Benefits and gains (2) Costs and losses and (3) Improving the cost-benefit ratio in order to prevail with the treatment. Participants described benefits in terms of an advanced circadian phase, improved everyday lives and self-evaluation. However, adhering to treatment and the resulting circadian phase advance also involved certain costs and losses. Still, participants also described how cost could be reduced by gradually tailoring the treatment protocol and their daily routines to individual needs.

**Conclusion:**

All participants considered the treatment to be worth the effort, but individual tailoring was necessary to minimize the effort/cost. The benefits of the treatment extended beyond sleep and circadian phase, positively affecting self-evaluation and beliefs regarding others' perception.

## Introduction

Delayed Sleep-Wake Phase Disorder (DSWPD) is a circadian rhythm sleep-wake disorder, characterized by a delay of the endogenous sleep-wake rhythm in relation to the desired or socially required sleep-wake timing, to such extent that it causes chronic or recurrent inability to fall asleep and difficulties waking up at socially acceptable times (American Academy of Sleep Medicine, 2023). The prevalence is reported to be between 1.1% and 8.4% (American Academy of Sleep Medicine, 2023). Consequences of DSWPD depend on whether the individual is able to rise at acceptable times. Whereas early rise times may cause daytime impairments both due to sleep loss and to being awake during one's biological night, late rise times may have consequences in terms of educational or occupational unattendance and social isolation. Accordingly, DSWPD has shown to negatively affect several aspects of the lives of afflicted individuals, not only in terms of sleep but also regarding mood, health behaviors, daytime performance, self-evaluation and social life (Pallesen and Bjorvatn, 2021; Saxvig et al., 2012; Wilhelmsen-Langeland et al., 2012). A recent meta-analysis showed that DSWPD is associated with a greater severity of depressive symptoms among young individuals (Dama et al., 2025).

Qualitative studies have elaborated on how DSWPD in adolescents affects family life and parental wellbeing (Montie et al., 2019), and that young adults with DSWPD experience social stigma related to their circadian rhythm and its sequalae (Wilhelmsen-Langeland et al., 2012).

Current treatment for DSWPD is based on well-known chronobiological principles, and usually involve individually timed bright light therapy and/or exogenous melatonin, often in conjunction with reduced evening light exposure and sleep scheduling (Meyer et al., 2022; Narala et al., 2024). Such treatment protocols have shown to effectively advance the sleep-wake pattern and improve daytime performance in individuals with DSWPD (Saxvig et al., 2014; Wilhelmsen-Langeland et al., 2013b). Still, researchers and clinicians have noted that DSWPD seems to be more difficult to treat than other sleep disorders, and that individuals with DSWPD often have problems adhering with the treatment (Micic et al., 2016). These difficulties may be attributable to chronobiological factors causing a rigidity in the circadian phase of individuals with DSWPD, which can make it extremely hard for them to adequately phase advance, even when all zeitgebers are optimally timed (Gomes et al., 2021). Although good acute effects of treatment are achieved, long-term therapy is often necessary to maintain treatment gains. Moreover, to follow the described treatment protocol requires time and effort, as well as a structuring of daily routines. Thus, motivational and psychological issues as well as personality traits may be crucial factors to address for treatment success (Micic et al., 2016). The fact that many individuals with DSWPD have experienced negative feedback from others because of their problem and therefore may consider themselves as lazy, may hinder persistence in following treatment protocols (Wilhelmsen-Langeland et al., 2012).

DSWPD is associated with low scores on the personality traits conscientiousness, extroversion and agreeableness, and high scores on neuroticism (Micic et al., 2017; Wilhelmsen-Langeland et al., 2013b). These tendencies may not only predispose to the disorder, but also comprise a particular challenge in terms of adherence to the required protocol. Some studies also suggest that DWSPD is associated with poorer executive functions than same-age peers, suggesting that following instructions and planning a schedule can be difficult (Wilhelmsen-Langeland et al., 2019).

Moreover, individuals with DSWPD often function very poorly in the morning, with higher awakening threshold, more sleep inertia and poorer cognitive function compared to their peers (Saxvig et al., 2019; Solheim et al., 2014, 2018). This may pose a challenge to treatment as morning routines are of particular importance when treating DSWPD. Unfortunately, it appears that many adolescents and young adults find the treatment regime challenging, as indicated by poor adherence to treatment (Gradisar et al., 2011) and high drop-out rates from studies in the ranges of 17%−40% (i.e., Gradisar et al., 2011; Mundey et al., 2005; Nagtegaal et al., 1998; Rosenthal et al., 1990).

In line with a systematic review pointing out the sparsity in knowledge about the reasons for adherence or non-adherence (Faulkner et al., 2020), we propose that a phenomenological understanding of these issues is of crucial importance to aid clinicians treating patients with DSWPD. No qualitative study has to date explored how adolescents and young individuals with DSWPD experience treatment. Thus, the aim of this qualitative study was to explore how individuals with DSWPD describe undergoing treatment, with a particular focus on cost/benefit evaluations.

## Methods

### Study setting and participants

As part of a comprehensive treatment study for youth with DSWPD, individual in-depth, semi-structured interviews were conducted after an open-label, 3-month follow-up trial with individually timed bright light (10.000 lux minimum exposure 30 min) and evening exogenous melatonin (3 mg). All participants were diagnosed with DSWPD based on the diagnostic criteria of the ICSD-2 (American Academy of Sleep Medicine, 2005) and they had been screened for sleep disorders other than DSWPD, moderate to severe psychopathology (assessed with SCID-I screening interview; First et al., 1997), somatic disorders or conditions assumed to affect sleep (i.e. migraine, B12 deficiency), serious somatic disorders (i.e. rheumatoid arthritis, diabetes), medications or treatments assumed to affect sleep (i.e. sedative antihistamines, antidepressants, hypnotics), substance abuse or night work. No participants had IQ <70 and none were breast feeding or pregnant (Wilhelmsen-Langeland et al., 2012). For more details on the study protocol and results, see Saxvig et al. (2014) and Wilhelmsen-Langeland et al. (2013a). The recruitment among college, university and high school students was done partly for practical reasons, partly because there was a scarcity of clinical studies on this patient group, and partly because DSWPD may have severe consequences for their learning and performance in their studies. The first author conducted the interviews from June 2009 until August 2011. The intention was to interview all 20 participants who received treatment in the 3-month follow-up trial. However, 2 participants dropped out of the treatment, 2 interviews were lost due to technical failure and 5 could not be interviewed since they completed the study while the interviewer was on maternity leave, leaving a total of 11 informants (see Table 1). Some of the participants had previously been informants in a pre-treatment qualitative study on psychosocial challenges related to DSWPD (Wilhelmsen-Langeland et al., 2012).

**Table 1:** Demographics for each informant including length of interview.

**Informant**	**Age**	**Gender**	**Educational level**	**Length of interview (min/sec)**
1	17	Male	High school student	17:00
2	23	Female	Student	43:00
3	24	Female	Student	48:30
4	23	Female	Student	27:53
5	25	Female	Student	22:00
6	24	Male	Employed	22:00
7	23	Female	Student	25:00
8	26	Male	Student	21:00
9	26	Female	Student	14:00
10	24	Male	Student	22:00
11	23	Female	Student	20:00

### Treatment protocol

The treatment administered before the interviews is described in previous publications (Saxvig et al., 2014; Wilhelmsen-Langeland et al., 2013a), summed up they were: instructed to sleep until spontaneous awakening on the first day of treatment, advance rise time by 1 h each day until the preferred rise time was reached (self-chosen), preferred rise time was then maintained throughout the treatment period. The participants were instructed to administer light therapy (10,000 lux) every day immediately upon awakening, for 30–45 min with eyes directed toward the lamp and to make sure that the distance to the lamp did not exceed 40–50 cm. In the evenings, 12 h after awakening, participants were to take a melatonin (3 mg) capsule. In the case of oversleeping, participants were to take light immediately upon awakening, melatonin 12 h later and to advance rise time with 1 h on the following days. No information was provided regarding bedtime.

A form to be completed each day of the 3-month treatment period was developed to measure adherence of treatment. Points were given for light exposure (1 point) and melatonin capsule taken (1 point). A full score on both, was rated as 100% adherent (Wilhelmsen-Langeland et al., 2013a).

### Ethics

Informed consent to participate in the trial was obtained after a comprehensive description of the study protocol. For participants 16–18 years of age, parents were required to sign the consent form before inclusion, as well as to verbally provide consent. None of the participants had previously received treatment for DSWPD, and none of them had been diagnosed with DSWPD prior to inclusion. All participants received a compensation fee (~€40) for their time invested in the full study. The study was approved by the Regional Committee for Medical and Health Research Ethics in Western Norway and by the Norwegian Social Data Service. The RCT was first registered in ClicialTrials.gov NCT00834886 at February 2nd 2009.

### Data collection and interview guide

The interview guide (see Supplementary file 1) was developed with an introductory request to rate whether they thought the treatment was worth the effort, from 0 (not worth the effort at all) to 100 (totally worth the effort). Further questions elaborated on the first response and a few probes were formed in advance based on the authors‘ clinical experiences of treating patients with DSWPD. All authors of this article contributed to the development of the interview guide; four psychologists (AWL, ID, SP, & IHN), one medical doctor (BB) and one physiologist with extensive experience within the sleep field (IWS). The wording in the interview guide was developed based on Kvale's (1994) recommendations for beginning with open questions to gain insight into the informants‘ life world, and then using a funneling approach where more specific questions were asked. During the interviews, the interviewer sought to check her understanding by reflecting back to the informant what she had heard and asking for descriptions of specific situations as opposed to general perceptions, also in line with recommendations by Kvale (1994). All interviews were audio taped (using a Sony Digital Recorder ICD-SX57) and transcribed verbatim by a psychology student/research fellow. In reporting the results, information from the context of the interview is inserted in square brackets when needed to ease the understanding of the citations. When parts of interviews have been removed, this is indicated by three dots in square brackets […]. To record adherence to treatment, informants were asked to register the number of days they took the melatonin capsule and used the light therapy lamp as prescribed.

### Analysis

The analysis followed the six steps of reflexive thematic analysis as described by Braun and Clarke (2006). The six steps are: (1) familiarizing with the data, (2) generating initial codes, (3) searching for themes, (4) reviewing themes, (5) defining and naming themes, and (6) producing a report. Initially, the first and last author (AWL and IWS) read and reread the transcribed data to become familiar with its content. The researchers were both female with different professional backgrounds, both having extensive experience in sleep research and experienced in terms of conducting qualitative research studies. They individually coded the data and further organized the identified codes. Thereafter, AWL and IWS met for a workshop to sort the codes into a range of possible aspects and patterns and discussed how different codes may be combined to form more overarching themes. The themes were reviewed and renewed in step four. The analysis team (AWL, IWS and ID) then defined the final themes and agreed on their essence. In the final analysis step, the themes were clearly defined and named. The analysis was assisted by the NVivo software (QSR International).

## Results

For the introductory request, the mean rating was 72.5 (range 60–100), indicating that all informants considered the treatment benefit to outweigh the effort (all ratings > 50). Most participants described the treatment as being helpful. Many had already bought a light therapy lamp and arranged for a melatonin-prescription with their doctors, while others said they would only continue or re-engage in treatment if they were more severely affected by DSWPD. Interestingly however, despite the initial > 50 rating, a few informants said they would never go through such treatment again, that it was too much hassle, even though it seemed to have benefits.

Self-reported adherence to treatment, defined as (1) taking the melatonin capsule at the prescribed time and (2) exposure to light therapy lamp a minimum of 20 minutes as prescribed ranged from 7 % to 100 % (average 64.4 %). The number of days participants took the melatonin capsule (possible range from 0 to 90) averaged 62.5 days, and the number of days they used the light therapy lamp as prescribed (a minimum of 20 min) ranged from 8 to 90, average 54.1 days.

In the text we use the words *most, many*, and *a few*. Most means six or more, many means four or more and a few means three or less.

### Themes

The qualitative analysis was organized according to three themes, which we named: (1) Benefits and gains; (2) Costs and losses; and (3) Improving the cost-benefit ratio in order to prevail with the treatment.

#### Theme 1: benefits and gains

Participants described falling asleep faster or easier than before. One participant said that she managed to deliberately “go to bed” as opposed to falling asleep from exhaustion: “*I used to fall asleep because of exhaustion while now I sort of go to bed to go to sleep”* (Informant 4). Being able to sleep at night could feel as a relief: “*It is just… I was just too sick of… sitting up until five in the morning, or four-five… and be tired during the day when everybody else are awake”* (Informant 7). Informant 2 said: “*So… just being able to go to bed and not be afraid of not being able to fall asleep… is very nice […].”* Informant 2 had felt discouraged before attending the intervention, almost having resigned to her delayed sleep problems as permanent. She was enthusiastic about the changes she experienced and wondered if her cognitive functioning had improved as a result:

“And… yes, it is just so lovely to have a good night sleep (laughs) […] or, I had almost given up a bit... that I had to live with it (laughs)… but eh… now it is just a whole new life […] and then I think maybe that my memory has improved.” (Informant 2)

Informant 2 continued explaining: “*I have felt sharper […] I feel like I pay more attention, things [at school] are less boring. […] I get better grades”*. Likewise, another participant described being more awake and able to attend long class exercises:

“I have functioned very well when I have arrived… for once early, at school […] and I have been able to work for 12 h if I arrive early enough […] now this [ability] is much more stable, it's no longer that I stay [doing academic work] only 4 hours one day and another day twelve.” (Informant 10)

The intervention called for structured routines, and participants described the intervention as being “disciplining”. Formerly, participants could stay up late to do their homework at night, working alone: “*So if I didn't get up until one in the afternoon, and had to study until ten at night, then there were no one to do stuff with… people went to bed [laughs]”* (Informant 2). One participant described being surprised to discover that having routines had some advantages: “*eh I was very skeptical… skeptical in the beginning but eh... I have realized eventually that there are... advantages of having a steady routine”* (Informant 10). Using the bright light was described as providing time for more satisfying morning routines: “*I have even time to take a shower and eat breakfast and watch the morning TV-show and… talk to my roommate before I go to school”* (Informant 4).

Participants also described other benefits from the intervention. One benefit was to become more in tune with the societal rhythm of people around them. One informant described the satisfaction of having developed the same rhythm as her roommates: “*And I now have the same circadian rhythm as my roommates too, so that is very practical” (*Informant 4). Many mentioned being able to attend school, as opposed to oversleeping, and as a result also becoming part of the social network at school: “*I have... in a way… become a part of the class more too, because I actually am at school”* (Informant 2).

Importantly, the changes in circadian rhythm changed participants' evaluation of themselves for the better, as well as their beliefs about how others perceived them. They seemed to have moved away from considering themselves as lazy, explaining their former problems differently than before:

“There is really quite a lot that has changed, and not the least how others view me […] Now it is like wow! You are at school! (laughs) […] I feel better… ehm… I feel a bit more successful (laughs) […] And I sort of feel as good as everybody else.” (Informant 2)

Some of the participants made dramatic changes in their lives, becoming more physically active and eating more healthily. They reasoned that these changes followed naturally from no longer being up all night as well as changing their eating habits: “*I mean it is logical you know, more controlled eating, not up all night… leads to more energy… I have reduced my habit of eating kebab and all sorts of funny food… it is quite nice”* (Informant 4). Informant 4 stated that the intervention had led to more energy and time to visit the gym, which enabled walking quickly in stairs instead of sluggishly like before. She claimed the protocol even helped her lose weight: “*I lost 10 kilos.”* Another informant said:

“I used to think maybe more that… [I] leaned more toward [the explanation] that, I may, well, then I must be a bit sort of a lazy person who… or a type b-personality who likes to sleep in and yeah, well then that is how I am, right? […] I have a bit of a different view of it now.” Informant 3

Informant 5 believed that she appeared different to others, after the intervention: “*I…. seem in a bit better mood in a way and a bit fresher and… yeah”* (Informant 5).

#### Theme 2: costs and losses

The treatment protocol itself was described as costly in terms of time and effort. Informant 1 noted: “*It is in a way a huge effort, time-consuming, to have to get up half an hour earlier to sit in front of that light.”* Informant 3 had a similar view: “*What was the hardest was kind of… [to] set aside enough time in the morning to also use the light for a long period of time.”* Having problems adhering with the protocol could be disappointing:

“… but then I haven‘t been able to go through with the treatment like really… and that is the reason that I... that it is not more than seventy percent… I haven‘t in a way… haven't improved myself as much as I had hoped for.” (Informant 5)

Disappointment could also occur when participants' circadian rhythm slid back to being delayed after the treatment ended. Informant 10 explained: “*And it has, it has gone gradually downwards I didn‘t understand that the effect was so clear until it wasn‘t there anymore*.” Participants also described a sense of loss, when making a change to a more common and accepted circadian rhythm. For example, they would lose valuable time they formerly had available in the evening. Informant 10 noted*: “I have been able to get a lot done in the afternoon-evening usually… […] and eh I have gotten used to that way of living,”* while Informant 4 noted: “*what I miss… is that before, if I had a big assignment, I just stayed up for 24 h and worked all through the night … I can't do that anymore […] But now I am tired around eleven at night (laughs).”*

#### Theme 3: improving the cost/benefit ratio in order to prevail with the treatment

The third theme comprises participants' views about how the cost/benefit ratio and their views of how this could be improved so they would be able to prevail with the intervention. Prevailing with the treatment was an issue for several participants. Although costs in time and effort could be high, they were sometimes explicitly described as worth the benefit, implying that prevailing was important: “*I felt, right, that I got back the effort I put in… in a way it was not so much to do but I got… everything in return for it”* (Informant 9). Other participants seemed to sense that costs were high, and if they had been more severely affected by DSWPD, they might have been more motivated to follow the treatment protocol. Most informants expressed an intention to continue treatment after the study ended, albeit using a modified version of the protocol, for example using either only melatonin, only light therapy or only strict sleep scheduling. Others described that knowing what to do to advance the sleep/wake pattern was sufficient for their purpose, and that they would use the lamp and melatonin more systematically when necessary (but not all the time).

Participants described efforts to be aware of which parts of the intervention that was least costly for them personally and adhere with those. A few described the routine of taking the melatonin capsules as easier to adhere to than the light therapy, informant 1 said: “*Yes, capsules were kind of not, they were kind of not a problem… they are not time consuming, that is just the light [which is time consuming].”* Most commonly informants described the light as their favorite part of the treatment protocol, described by informant 2 like this: “*If I were to choose one thing, I think it would be just the light… It is because I have gotten so fond of it (laughs)… But I like being able to get out of bed and be alert*.”

One participant described how he at first thought it would be an extreme effort to carry out the treatment protocol. However, he forced himself to do it. After a while the routines became an automated habit. He compared this to adhering with other routines with little effort, such as taking the bus to school, and reasoned that following the treatment protocol could be just as manageable:

“*… but I don‘t know, it was, I just knew, realized… I had to take one half hour of light every morning, I just knew it would be a crazy effort… But in the beginning, you just have to force yourself to... do it… because as soon as it becomes automated, it is fine… I mean, I take the bus to school every morning, that is no effort for me at all.”* (Informant 1)

The gradual realization of improvements was described as increasing the motivation to continue. This could happen already after 1 week, informant 2 experienced: “*In the first 2 weeks when I wasn‘t used to rise early… it is of course... But ehhh… after a week I think I noticed that the light was helping or like that… that I felt a bit more alert, after having looked into the light*.” Another participant described changes after 3–4 weeks:

“Yes… and then in the last two-three, three-four weeks now that I have woken up by myself (with no alarm clock) at around eight-nine o‘clock during the day, now in the morning… So… I have had even greater improvement (Interviewer asks how long it took before he/she noticed any change). Maybe... three weeks, three-four weeks.” (Informant 7)

One participant suggested that it would have been easier to follow the protocol, if one could find technical solutions that would enable people to follow the routines while going about their daily activities. Informant 7 suggested designing a portable lamp: “*Yes, like a helmet with the light on it... do they exist? [laughs].”*

## Discussion

Treatment with individually timed bright light, exogenous melatonin and sleep scheduling has demonstrated to effectively advance the sleep-wake pattern in patients with DSWPD. Still, to our knowledge, no previous study has utilized qualitative methods to explore how patients with DSWPD experience this kind of treatment. Through in-depth interviews with adolescents and young adults who had received treatment for DSWPD in a 3-month, open label trial, we formulated three themes: (1) Benefits and gain; (2) Costs and loss; and (3) Improving the cost-benefit ratio in order to prevail with the treatment.

### Benefits and gains

Benefits were described in terms of direct effects of treatment: finding it easier to fall asleep, feeling more alert during the day and experiencing advantages of structure and routines. There were also indirect benefits of treatment effects such as better alignment with the social environment, improved view of self and feeling that they were more favorably perceived by others. Informants described being able to go to bed and fall asleep after the treatment, having accumulated sufficient sleep pressure.

Our clinical experience is that being unable to fall asleep when going to bed, and not feeling sleepy when others go to bed, are common complaints amongst those with DSWPD. This may explain why many adolescents afflicted with DSWPD do not have common bedtime routines, they just do not expect to fall sleep at an acceptable time. Establishing a bedtime routine may follow spontaneously as the sleep-wake patterns shift toward earlier bedtimes. Informants described gains in terms of improved daytime alertness, more school attendance and a greater ability to be attentive and awake at school, even finding school less boring. This is encouraging since patients with DSWPD often perform worse at school compared to same-age peers, and DSWPD involves an increased risk of unemployment (Danielsson et al., 2016a; Futenma et al., 2023).

Many informants described experiencing their new routines as beneficial and important. Being able to rise early resulted in more spare time in the afternoon when they also could socialize. As mentioned, studies have indicated that DSWPD is associated with certain personality traits, particularly low conscientiousness (Micic et al., 2017; Wilhelmsen-Langeland et al., 2013b). To our knowledge, no studies have to date investigated the trajectory of DSWPD and personality development. Future studies should specifically address the relationship between DSWPD-symptoms and personality traits by longitudinal designs and in treatment studies. It is natural that after years of receiving advice on what to do regarding sleep, and trying to follow this advice without success, influences the way the person views him/herself. It is further recommended to focus more on individually optimized treatment that also addresses psychological factors as well as the lifestyle and environment of young people (Futenma et al., 2023).

### Costs, losses, and the perceived cost/benefit ratio

Costs were both related to the treatment protocol itself, particularly the light therapy (time and effort), which is in line with what was found in a systematic review (Faulkner et al., 2020), but our informants also described treatment failures (disappointments) and treatment effects (e.g. losing valuable wake times in the evening/night). When asked if the treatment was worth the effort, the ratings ranged from 60 to 100, indicating that overall, all informants considered the benefits to outweigh the costs. Many of the informants reflected on how costs could be reduced, and how adaptations of the protocol could improve the cost/benefit ratio. The suggestion by informant 7 about a “helmet with the light on”, does exist in the shape of visors or glasses with light directed toward the eyes (i.e., Feel Bright Light, 2020; Luminette Lucimed SA, 2015; Re-timer, 2010). These devices may alleviate the treatment protocol in regard to the time it takes to sit stationary in front of a bright light lamp, as some morning routines can be done while wearing visors or glasses with light.

Most informants expressed an intention to continue treatment after the study ended, using a modified version of the protocol, for example using either only melatonin, only light therapy or only strict sleep scheduling. Others said that knowing what to do to advance the sleep/wake pattern was sufficient and would use the tools that had been provided during the intervention if it turned out to be necessary later on. A few of the informants pointed out the need to understand the intervention better and the need for having consult during the treatment. Feeling more in control of their sleep and circadian rhythm might reduce feelings of helplessness and failure and may reduce symptoms of depression and “feeling different”. As most participants were students with teaching/classes during daytime they may have benefited more from the treatment than groups with less time constraints (e.g., people working freelance).

### Strengths and limitations

#### Strengths

The study protocol was derived from an experienced research team with both clinical and sleep research expertise. It also comprised researchers experienced in conducting qualitative research. This facilitated discussions from a wide range of perspectives, enabling a nuanced understanding of the interviews. The interviews were performed by a clinical psychologist with experience from both qualitative studies and treatment for DSWPD (Wilhelmsen-Langeland et al., 2012) enabling in-depth explorations facilitated by clinical expertise and training. The themes identified in the study were reflected by statements by many interviewees. Although the number of respondents endorsing a specific theme does not necessarily make the findings more valid than themes less extensively covered (Braun and Clarke, 2006, 2019), it does by some other standards contribute to the internal validity of the study (Morse, 2015).

Adherence was registered for the total group of youth receiving 3-month treatment and thirteen participants (65%) were rated as adherent to treatment (≥50%) and 7 (35%) as non-adherent (<50%) (Wilhelmsen-Langeland et al., 2013a).

#### Limitations

The transferability of the results to other patients with DSWPD presenting, for example, in a physicians' clinical practice, is difficult to ascertain. The informants in this study had first undergone a two-week treatment and were then re-randomized to treatment with light therapy + melatonin 3 mg for 3 months, hence they had received the instructions twice and tried the treatment twice. That might mean that they had more information and were better prepared for the three-month treatment phase than a person who would turn up at their medical doctors‘ office presenting with DSWPD. Also, a limitation is that the participants in this study were part of a large RCT and had accepted a quite comprehensive plan to participate. They may thus have been a selected group and not representative of most DSWPD sufferers. Moreover, our sample was mainly recruited from high school and colleges/university, hence those who already have dropped out from such education were not recruited. Although only 11 participants were included in the study, the study is high in “information power” (Malterud et al., 2016). Participants were purposefully sampled for their rich and relevant experience with the research topic; the interviewer had the necessary clinical and research background to pose relevant follow-up questions; and the study aim was narrow and specific. These aspects all contribute to the study's information-power as noted by Malterud et al. (2016). We did not interview the two participants who dropped out; hence we lack their perspective which might have diverged from the others.

### Implications for research and clinical practice

We believe that it is of crucial importance to disseminate information about DSWPD and the possible gains of early intervention. The costs described by the informants in this study are understandable reasons for adherence or non-adherence and need to be taken seriously. Patients may need information that hassles, for example those associated with the lamp, typically are reduced with time. They may need to know that there is commonly a change in the sleep-wake pattern in the beginning of this treatment, that may be problematic, but as the circadian phase advances both at wake-up time and at the time of falling asleep, it gets better. Still, some losses will become permanent if they prevail the treatment, such as reduced ability to work at night and have their “quiet and creative” solitary time when everybody else is asleep. Informing patients early on that this is a treatment they will need to uphold rather than a cure, is probably of great importance.

In line with Faulkner et al. (2020), this study elaborates on how a reconceptualization of light interventions as complex behavior change interventions is called for. Individuals want to tailor treatments to their preferences, and that tailoring of treatment for DSWPD should be a conjoint activity between treatment provider and patient. Patients need to know that they may tailor aspects of the protocol according to their individual preferences, but also which components of the protocol are essential in order to receive benefit. To which extent the treatment protocol can be adapted to patient preferences, is a difficult question that calls for further studies. However, knowing more about patient preferences and needs may bring us one step closer to finding the balance between medical requirements and patient possibilities. Such understandings are valuable when working to establish a solid working alliance between treatment provider and adolescent patients, especially regarding DSWPD. Viewing treatment for DSWPD as a process, where helpers are flexible and able to adjust the treatment protocol as necessary, may increase the likelihood of success.

We believe it is important to discuss possible pros and cons of treatment with patients suffering from DSWPD before they initiate treatment, for example by asking them if they can make space in their schedules. In cases where one has developed a strong negative self-evaluation or psychiatric comorbidities, psychotherapy or more advanced help may be necessary in addition to light therapy, sleep scheduling and possibly exogenous melatonin. Group-formats with relaxation techniques and cognitive restructuring have proven to be appreciated by patients with DSWPD (Jansson-Fröjmark et al., 2016) and may be beneficial for some patients with DSWPD. Kaplan et al. (2019) concluded that light exposure during sleep (3-millisecond light flash every 20 seconds during the last hours of sleep) in combination with a motivation-focused CBT intervention advanced bedtimes and increased total sleep time in adolescents with difficulties going to bed earlier and waking up early enough.

In order to avoid overtreatment, we suggest a stepped care-model, beginning with a clear description of the physiology behind the disorder and the sleep scheduling protocol with light therapy and/or melatonin depending on patient preference. If in need of more follow-up, a more elaborate treatment regime may be useful. Finally, being informed that it is their chronobiology that is out of sync with the societal rhythm, rather than blaming it on their behavior or their choices, may increase treatment motivation.

### Conclusion

All participants in this study considered the treatment to be worth the cost, but many participants described that they initiated individual tailoring to minimize the effort and to overcome barriers for adherence. The benefits of the treatment extended beyond sleep/circadian phase, positively affecting self-evaluation and beliefs regarding others' perceptions of them. The study adds to the knowledge base on how to develop practical and effective treatment approaches to treat DSWPD and to how to deal with adherence. The information provided through this study, also adds to the knowledge about the consequences of DSWPD for those affected and their surroundings. With this information, we are one step closer to being able to develop improved and individualized treatment plans that are patient-centered and user-developed.

## Data Availability

The raw data supporting the conclusions of this article will be made available by the authors, without undue reservation.

## References

[B1] American Academy of Sleep Medicine (2005). The International Classification of Sleep Disorders: Diagnostic and Coding Manual (2nd ed.). Darien, IL: American Academy of Sleep Medicine.

[B2] American Academy of Sleep Medicine (2023). International Classification of Sleep Disorders (3rd ed, text revision). Darien, IL: American Academy of Sleep Medicine.

[B3] Braun V. Clarke V. (2006). Using thematic analysis in psychology. Qual. Res. Psychol. 3, 77–101. 10.1191/1478088706qp063oa

[B4] Braun V. Clarke V. (2019). Reflecting on reflexive thematic analysis. Qual. Res. Sport, Exerc. Health 11, 589–597. 10.1080/2159676X.2019.1628806

[B5] Dama M. H. Martin J. Tassone V. K. Lin Q. Lou W. Bhat V. (2025). The Association between delayed sleep-wake phase disorder and depression among young individuals: a systematic review and meta-analysis: association entre le syndrome de retard de phase et la dépression parmi les jeunes: revue systématique et méta-analyse. Can J. Psychiatry. 1–18. 10.1177/07067437251328308 40129277 PMC11948252

[B6] Danielsson K. Jansson-Fröjmark M. Broman J. Markström A. (2016a). Cognitive behavioral therapy as an adjunct treatment to light therapy for delayed sleep phase disorder in young adults: a randomized controlled feasibility study. Behav. Sleep Med. 14, 212–232. 10.1080/15402002.2014.981817 26244417

[B7] Faulkner S. M. Dijk D. J. Drake R. J. Bee P. E. (2020). Adherence and acceptability of light therapies to improve sleep in intrinsic circadian rhythm sleep disorders and neuropsychiatric illness: a systematic review. Sleep Health 6, 690–701. 10.1016/j.sleh.2020.01.014 32173374

[B8] Feel Bright Light (2020). Feel Bright Light. Available online at: https://feelbrightlight.com/ (accessed April 04, 2025).

[B9] First M. B. Spitzer R. L. Gibbon M. Williams J. B. (1997). User‘s guide for the structured clinical interview for DSM-IV axis I disorders. SCID-I. Clinical Version. American Psychiatric Publishing, Inc.21156992

[B10] Futenma K. Takaesu Y. Komada Y. Shimura A. Okajima I. Matsui K.. (2023). Delayed sleep-wake phase disorder and its related sleep behaviors in the young generation. Front. Psychiatry 14, 1174719. 10.3389/fpsyt.2023.1174719 37275982 PMC10235460

[B11] Gomes J. N. Dias C. Brito R. S. Lopes J. R. Oliveira I. A. Silva A. N.. (2021). Light therapy for the treatment of delayed sleep-wake phase disorder in adults: a systematic review. Sleep Sci 14, 155–163. 10.5935/1984-0063.20200074 34381579 PMC8340891

[B12] Gradisar M. Dohnt H. Gardner G. Paine S. Starkey K. Menne A.. (2011). A randomized controlled trial of cognitive-behavior therapy plus bright light therapy for adolescent delayed sleep phase disorder. Sleep 34, 1671–1680. 10.5665/sleep.1432 22131604 PMC3208844

[B13] Jansson-Fröjmark M. Danielsson K. Markström A. Broman J. E. (2016). Developing a cognitive behavioral therapy manual for delayed sleep-wake phase disorder. Cogn. Behav. Ther. 45, 518–532. 10.1080/16506073.2016.1207096 27454077

[B14] Kaplan K. A. Mashash M. Williams R. Batchelder H. Starr-Glass L. Zeitzer J. M. (2019). Effect of light flashes vs sham therapy during sleep with adjunct cognitive behavioral therapy on sleep quality among adolescents: a randomized clinical trial. JAMA Netw Open 2, e1911944. 10.1001/jamanetworkopen.2019.11944 31553469 PMC6763980

[B15] Kvale S. (1994). Interview - En introduksjon til det kvalitative forskningsinterview. Copenhagen: Hans Reitzels Forlag.

[B16] Luminette Lucimed SA (2015). Luminette Light Therapy Device. Villers-le-Bouillet: Lucimed SA. Available online at: https://myluminette.com (accessed April 04, 2025).

[B17] Malterud K. Siersma V. D. Guassora A. D. (2016). Sample size in qualitative interview studies: guided by information power. Qual. Health Res. 26, 1753–1760. 10.1177/1049732315617444 26613970

[B18] Meyer N. Harvey A. G. Lockley S. W. Dijk D.-J. (2022). Circadian rhythms and disorders of the timing of sleep. Lancet 400, 1061–1078. 10.1016/S0140-6736(22)00877-7 36115370

[B19] Micic G. Lovato N. Gradisar M. Burgess H. J. Ferguson S. A. Lack L. (2016). The etiology of delayed sleep phase disorder. Sleep Med. Rev. 27, 29–38. 10.1016/j.smrv.2015.06.004 26434674

[B20] Micic G. Lovato N. Gradisar M. Lack L. C. (2017). Personality differences in patients with delayed sleep-wake phase disorder and non-24-h sleep-wake rhythm disorder relative to healthy sleepers. Sleep Med. 30, 128–135. 10.1016/j.sleep.2016.04.002 28215235

[B21] Montie K. Quaedackers L. Perlitius V. van der Horst E. Vandenbussche N. Overeem S.. (2019). The impact of delayed sleep phase disorder on adolescents and their family. Sleep Med. 64, 15–22. 10.1016/j.sleep.2019.05.022 31655320

[B22] Morse J. M. (2015). Critical Analysis of Strategies for determining rigor in qualitative inquiry. Qual. Health Res. 25, 1212–1222. 10.1177/1049732315588501 26184336

[B23] Mundey K. Benloucif S. Harsanyi K. Dubocovich M. L. Zee P. C. (2005). Phase-dependent treatment of delayed sleep phase syndrome with melatonin. Sleep 28, 1271–1278. 10.1093/sleep/28.10.1271 16295212

[B24] Nagtegaal J. E. Kerkhof G. A. Smits M. G. Swart A. C. Van Der Meer Y. G. (1998). Delayed sleep phase syndrome: a placebo-controlled cross-over study on the effects of melatonin administered five hours before the individual dim light melatonin onset. J Sleep Res. 7, 135–143. 10.1046/j.1365-2869.1998.00102.x 9682186

[B25] Narala B. Ahsan M. Ednick M. Kier C. (2024). Delayed sleep wake phase disorder in adolescents: an updated review. Curr. Opin. Pediatr. 36, 124–132. 10.1097/MOP.0000000000001322 38054481

[B26] Pallesen S. Bjorvatn B. (2021). “Circadian rhythm sleep disorders. Health risks,” in Sleep Medicine Text Book, eds. W. M. B. Bassetti, T. Paunio, & P. Peigneux (2.nd ed) (Regensburg: European Sleep Research Society), 483–492.

[B27] Re-timer (2010). Re-timer Light Therapy Device. Available online at: https://www.re-timer.com (accessed April 04, 2025).

[B28] Rosenthal N. E. Joseph-Vanderpool J. R. Levendosky A. A. Johnston S. H. Allen R. Kelly K. A.. (1990). Phase-shifting effects of bright morning light as treatment for delayed sleep phase syndrome. Sleep 13, 354–361. 2267478

[B29] Saxvig I. W. Pallesen S. Wilhelmsen-Langeland A. Molde H. Bjorvatn B. (2012). Prevalence and correlates of delayed sleep phase in high school students. Sleep Med. 13, 193–199. 10.1016/j.sleep.2011.10.024 22153780

[B30] Saxvig I. W. Wilhelmsen-Langeland A. Pallesen S. Nordhus I. H. Vedaa O. Bjorvatn B. (2019). habitual sleep, social jetlag, and reaction time in youths with delayed sleep-wake phase disorder. a case-control study. Front. Psychol. 10:2569. 10.3389/fpsyg.2019.02569 31781012 PMC6861448

[B31] Saxvig I. W. Wilhelmsen-Langeland A. Pallesen S. Vedaa O. Nordhus I. H. Bjorvatn B. (2014). A randomized controlled trial with bright light and melatonin for delayed sleep phase disorder: effects on subjective and objective sleep. Chronobiol. Int. 31, 72–86. 10.3109/07420528.2013.823200 24144243

[B32] Solheim B. Langsrud K. Kallestad H. Olsen A. Bjorvatn B. Sand T. (2014). Difficult morning awakening from rapid eye movement sleep and impaired cognitive function in delayed sleep phase disorder patients. Sleep Med. 15, 1264–1268. 10.1016/j.sleep.2014.05.024 25156750

[B33] Solheim B. Olsen A. Kallestad H. Langsrud K. Bjorvatn B. Gradisar M.. (2018). Cognitive performance in DSWPD patients upon awakening from habitual sleep compared with forced conventional sleep. J Sleep Res. 28:e12730. 10.1111/jsr.12730 30105851

[B34] Wilhelmsen-Langeland A. Dundas I. Saxvig I. W. Pallesen S. Nordhus I. H. Bjorvatn B. (2012). Psychosocial challenges related to delayed sleep phase disorder. Open Sleep J. 5, 51–58. 10.2174/1874620901205010051

[B35] Wilhelmsen-Langeland A. Saxvig I. W. Johnsen E. H. Marszalek M. A. Wiig Andersen M. I. Saetre V. K.. (2019). Patients with delayed sleep-wake phase disorder show poorer executive functions compared to good sleepers. Sleep Med. 54, 244–249. 10.1016/j.sleep.2018.10.035 30590307

[B36] Wilhelmsen-Langeland A. Saxvig I. W. Pallesen S. Nordhus I. Vedaa Ø. Lundervold A. J.. (2013a). A randomized controlled trial with bright light and melatonin for the treatment of delayed sleep phase disorder: effects on subjective and objective sleepiness and cognitive function. J. Biol. Rhythms 28, 306–321. 10.1177/0748730413500126 24132057

[B37] Wilhelmsen-Langeland A. Saxvig I. W. Pallesen S. Nordhus I. Vedaa Ø. Sørensen E.. (2013b). The personality profile of young adults with delayed sleep phase disorder. Behav. Sleep Med. 12, 481–492. 10.1080/15402002.2013.829063 24283705

