# Supplementary material for: How do youth with Delayed Sleep-Wake Phase Disorder experience a chronobiological treatment protocol? An interview study
Source: Front Sleep. 2025 Jun 13;4:1555160. doi: 10.3389/frsle.2025.1555160 (PMC12713934; doi:10.3389/frsle.2025.1555160)
Supplement: Supplementary file 1 [file Data_Sheet_1.PDF]

<u>**Interview guide**</u>

Research question: Do you experience the treatment as worth the effort?

On a scale from 0-100, how much du you think the treatment was worth the effort?

(Explore further around this question)

Follow-up questions can be for example:

- Why did you not say a lower number?
- What would it take for you to say a higher number?
- Do you have suggestions for what could be done differently for the treatment top be more worth the effort?
- What do you think makes you say it was worth it to the extent you said?
- What changes (if any) have you noticed because of the treatment?

About the future

- Do you wish to continue the treatment? (explore around this)

Følsomhet Intern (gul)
